# Scatter-hoarding birds disperse seeds to sites unfavorable for plant regeneration

**DOI:** 10.1186/s40462-022-00338-1

**Published:** 2022-09-17

**Authors:** Marjorie C. Sorensen, Thomas Mueller, Isabel Donoso, Valentin Graf, Dominik Merges, Marco Vanoni, Wolfgang Fiedler, Eike Lena Neuschulz

**Affiliations:** 1 Senckenberg Biodiversity and Climate Research Centre, Frankfurt, Germany; 2 Kwantlen Polytechnic University, Surrey, Canada; 3 Department of Biological Sciences, Goethe University Frankfurt, Frankfurt, Germany; 4 Mediterranean Institute for Advanced Studies IMEDEA (CSIC-UIB), 07190-Esporles, Mallorca, Balearic Islands Spain; 5 Swedish University of Agricultural Sciences, Uppsala, Sweden; 6 Amt für Wald und Naturgefahren Graubünden, Chur, Switzerland; 7 Department of Migration, Max Planck Institute of Animal Behaviour, Radolfzell, Germany

**Keywords:** Spotted nutcrackers, GPS tracking, Seed dispersal, Ecosystem services, *Nucifraga caryocatactes*, *Pinus cembra*

## Abstract

Scatter-hoarding birds provide effective long-distance seed dispersal for plants. Transporting seeds far promotes population spread, colonization of new areas, and connectivity between populations. However, whether seeds transported over long distances are deposited in habitats favorable to plant regeneration has rarely been investigated, mainly due to methodological constraints. To investigate dispersal patterns and distances of Swiss stone pine (*Pinus cembra*) seeds we utilized advances in tracking technology to track the movements of their sole disperser, the spotted nutcracker (*Nucifraga caryocatactes*). We found routine individual movements between single seed harvesting and seed caching site. Harvesting sites of individual birds overlapped, whereas seed caching sites were separated and located on average 5.3 km away from the harvesting site. Interestingly, most distant caching sites were located at low elevations and in spruce forest, where Swiss stone pine does not naturally occur. This suggests that nutcrackers disperse seeds over long distances but that a large portion of these seeds are cached outside the known pine habitat. Therefore, we conclude that the implications of such long-distance seed dispersal movements for plant populations should be carefully considered in combination with the effects of habitat quality on plant recruitment.

**Supplementary Information:**

The online version contains supplementary material available at 10.1186/s40462-022-00338-1.

## Background

Seed dispersal is an important component of plant life history because it lays the spatial template for the population processes that follow. For animal-dispersed plants, an understanding of disperser movement behavior is critical to identifying spatial seed dispersal patterns and for making predictions regarding the population dynamics and distribution of plant species [21]. However, linking movement behavior to ecological processes, such as long-distance seed dispersal is a daunting task as most dispersers passively drop seeds in the environment [2, 8]. Previous studies have, for instance, combined movement data with gut passage times to estimate seed dispersal locations [20, 29, 30, 47]. Spatial biases in the accumulation of dispersed seeds at specific locations, so called directional seed dispersal [39], have been demonstrated for a number of plant-seed disperser interactions (e.g., [7, 8, 17, 38]). However, directional dispersal of seeds is not equivalent to *directed dispersal* in the strict sense, i.e., dispersal of seeds to habitat favorable for plant establishment [39, 46]. To date, it remains challenging to disentangle the spatial extent of seed deposition and its relation to plant recruitment [1].

Scatter-hoarding animals, which cache seeds under the soil surface for later consumption, are particularly suitable to study seed dispersal, as they actively deposit seeds at particular sites [34], which can often be explicitly identified, in contrast with passive animal-mediated seed dispersal (i.e., endozoochory). Scatter-hoarders provide especially effective seed dispersal as they bury seeds [43], which increases the chance of germination and protects seeds from predation by seed predators [4, 45]. The majority of data on scatter-hoarding animals come from studies of rodents (e.g., [4, 19, 35], however, scatter-hoarding birds can disperse seeds over much greater distances [36, 37]. Such long-distance dispersal is particularly important for plant populations as it for instance maintains the genetic diversity of separated sub-populations and enables shifts in plant species distributions [5]. While long-distance dispersal of scatter-hoarding birds has thus been regarded as generally beneficial for the plant [17, 25], seeds dispersed over long distances could also be directed towards low-quality habitat where the probability of establishment is poor. However, this phenomenon has rarely been addressed, likely due to the overall challenges of measuring long-distance dispersal using movement, genetic, or stable isotope approaches. Linking deposited seeds with the mother plant via DNA barcoding requires impractical sampling effort over large areas [14, 15]. Stable isotope enrichment is another suitable method to track seed deposition across large spatial scales [7, 8], but usually only covers dispersal distances of a few hundred meters. GPS tracking technology that now allows even small-bodied animals to be tracked frequently and over longer time frames in combination with an understanding of potential seed fate can help to shed light on the implications of long-distance dispersal for plant populations.

We conducted fine scale spatiotemporal GPS tracking of spotted nutcracker (*Nucifraga caryocatactes*) movements during the autumn ripening season of the Swiss stone pine (*Pinus cembra*) to investigate pine seed dispersal distances and dispersal patterns. Swiss stone pines depend exclusively on spotted nutcrackers, year-round residents of the European Alps, to transport their seeds as they are the only animals that can remove entire seeds from the hard cone [27]. Dispersal events by other species such as nuthatches or woodpeckers are very rare [27] and secondary dispersal by rodents occurs rather exceptionally (pers. obs). When pine seeds ripen from August to September, nutcrackers harvest and then transport seeds to different caching locations. Seeds are stored below the soil surface and are fed on until new seeds begin to ripen the following year. Swiss stone pine populations occur across a narrow elevational gradient ranging from about 1,500–2,400 m [42]. At the lower elevational range limit, seedling recruitment probability is low due to high seed predation, high canopy cover, and drier conditions [28, 33], additionally, pines are outcompeted by other tree species at lower elevations, in particular Norway spruce (*Picea abies*). Swiss stone pine populations are declining and have become increasingly fragmented and threatened due to human activities [11, 42], but see [12]. Studying seed dispersal is therefore important to estimate the regeneration potential of the species.

We investigated the movement behavior of spotted nutcrackers to ask: (1) whether harvesting and caching sites can be inferred by movement data, (2) how far and widespread nutcrackers transport pine seeds, (3) whether long-distance dispersal events of pine seeds result in caching at high elevation sites known to be the most favorable for pine seed germination and recruitment [28, 33].

## Methods

### Study site and species

We studied spotted nutcracker movements in 2017 and 2018 in the eastern Swiss Alps, in a ~ 15km^2^ area surrounding Davos, Switzerland. There, Swiss stone pine populations are a foundational species forming the upper tree line at between 1850 to 2200 m a.s.l. [27]. Seed-bearing trees grow at elevations of up to 2150 m a.s.l. and highest pine densities are located at intermediate elevations within the elevational range [33]. At the valley bottoms, forests are dominated by Norway spruce (*Picea abies*) and European larch (*Larix decidua*), with low Swiss stone pine abundance.

### Capture and GPS tracking

Spotted nutcrackers begin harvesting when Swiss stone pine seeds ripen from August to October. To capture nutcrackers we erected 3–4 mist nets in the Flüela valley (4648′0.25″N, 954′15.38″E), where a large stand of Swiss stone pine trees is located, from Aug 3–31 in 2017 and from Aug 6–19 in 2018. Mist nets were placed in areas where significant nutcracker activity had been observed previously. Nutcrackers were caught passively and without the use of playback or decoys. All individuals were aged as either adults or juveniles according to the occurrence and shape of white tips on wing and covert feathers [40]. We selected adults for GPS tag deployment as adults are most likely to have previous seed caching experience and to display harvesting and caching movement patterns typical of the overall population. A juvenile tagged in 2018 remained at the harvesting site and moved over a small area (~ 0.5 km^2^) during 20 days of the harvesting and caching season (see Additional file 1: Fig. S1). A total of 20 adult nutcrackers (5 in 2017, 15 in 2018) were fitted with 5.5 g backpacks containing a GPS, remote data readout, and a VHF radio transmitter (PinPoint VHF-120, Lotek, Newmarket, ON). The backpacks were a maximum of 3% body weight (all tagged nutcrackers were > 182 g) and included sewn breakpoints that were designed to allow the backpack to fall safely from the bird after ~ 2 months. We used radio receivers and two element-H antennas to locate and approach birds, then once within range we downloaded the data from the data logger. Remote data download was attempted daily for each tagged bird. When birds could not be located in the Flüela valley, we visited the surrounding valleys to attempt to make contact with tagged birds. Of 20 tagged birds, three were not contacted again after tagging and four had fewer than five days of data. This could have been most likely because of tag failure or because birds permanently moved to a location further away than we were able to detect. This resulted in a total number of 12 tags with full datasets throughout the tracking period that were included in the analyses. GPS tags were programed to record one location every 15 min from 10:00 to 20:00 h. One tag was programmed to record one location every 6 min from 10:30 to 11:30 h. As data recorded by this tag were comparable to the 15-min intervals (see Additional file 1, tag 37 in Fig. S2), we included this tag in the analyses. GPS point recording began the earliest at 10:00, as harvesting activity was slower in the early morning (pers. obvs). We tested the accuracy of Lotek GPS tags by placing two tags at the capture site and recording 95 fixes. The average linear error was 12.4 m (range 0–71.7 m, SD = 9.5 m, 50% CI = 12.8 m, and 95% CI 13.8 m). Overall tracking yielded movement data between 8 and 36 days (mean = 23.6 days) from 12 spotted nutcrackers.

### Movement analysis

We identified frequently revisited sites, time spent within these areas (visit duration), and time since the last visit to the area using the R package *recurse* [3]. We drew a radius of 500 m around each GPS point, such that each point could be treated as a potential revisitation site. We then calculated the number of revisits at each site. For one individual (tag 21), we used a 300 m radius, as the dispersal distances were too small to apply a 500 m radius. To isolate the frequently revisited sites, we set a threshold of revisits. To do so, we plotted the density of revisits, and then selected the minimum density as the threshold between the rarely revisited and the frequently revisited sites. In the rare cases where there was more than one local minimum (i.e., tag 20, 24, 27), we set the threshold at the minimum with the largest percentage of maximum revisits (Additional file 1: Fig. S2).

Observations at our capture site in the Flüela valley confirmed intensive seed harvesting behavior (i.e., nutcrackers flying with cones and working on cones to remove seeds) and an extensive Swiss stone pine stand. While at distant frequently visited sites a few single nutcrackers were observed, no harvesting behavior was seen, and most trees were spruce with few Swiss stone pines observed. Additionally, nutcrackers observed in long flights down valley from the Flüela capture site had sublingual pouches, which are used for seed transport, full of seeds. Once frequently revisited sites were identified, we performed a spatial fuzzy cluster analysis to group these sites into two clusters and then compared the visit durations between clusters. To measure the size of revisited sites, we extracted 95% convex polygons of each cluster using the “adehabitatHR” package [6]. We also extracted the straight linear distance between sites. To obtain information on the habitat type of frequently revisited sites, we calculated the overlap of revisited sites with Swiss stone pine forest cover. To do so, we used maps provided by the “Amt für Wald und Naturgefahren Graubünden” showing the proportion of Swiss stone pine in overall forest cover. We calculated the overlap between revisited sites with forest area that contained at least 10% of Swiss stone pine coverage. Finally, we extracted the elevation above sea level of revisited sites. To test for significant differences in visit duration, area, overlap with Swiss stone pine forest and elevation of revisited sites, we fitted a separate generalized linear mixed effects model for each response variable, including tag ID as a random effect.

## Results

We identified two revisitation sites for each individual, with routine movements between sites roughly every 2 h (mean 5.5 trips, SE 0.34, range 3.8–7.24, between sites per daily 10-h tracking period). While harvesting and caching sites were originally identified using field observations (see methods for additional details), we found that harvesting and caching sites could also be distinguished solely by the amount of time that nutcrackers spent at each site. Nutcrackers spent about 1.5 times more time in harvesting than in caching sites (Fig. 2a; Estimate: 0.4, SE 0.09, t = 4.4, *p* = 0.001). While nutcrackers spent on average 1.4 h at harvesting sites per visit, they only stayed for an average of 39 min at caching sites.

**Fig. 1:**
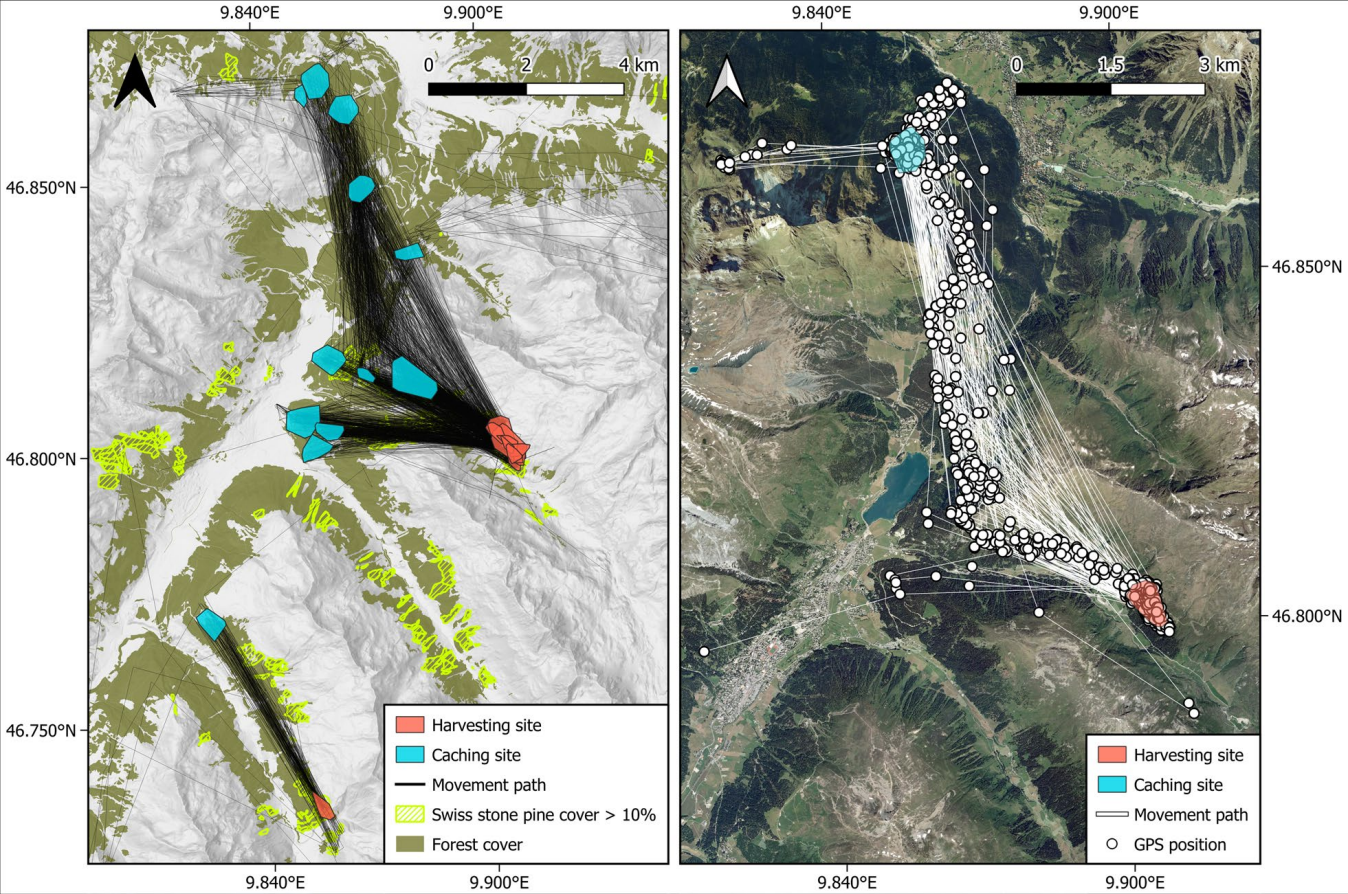
Map of the study area in Davos (Grison), Switzerland. Left: movements of 12 spotted nutcrackers (*Nucifraga caryocatactes*) during the harvesting season (August–September 2017 and 2018) of Swiss stone pine (*Pinus cembra*) seeds. Black lines: spotted nutcracker movements between harvesting (red minimum 95% convex polygon) and caching (blue minimum 95% convex polygon) sites. Right: movement path of one individual spotted nutcracker between harvesting and caching sites. White points denote GPS position every 15 min

**Fig. 2:**
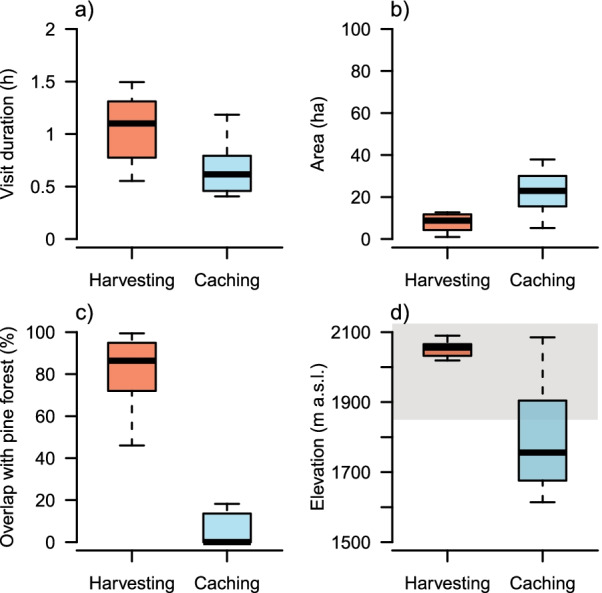
Differences between **a** visit duration, **b** area, **c** overlap with pine forest cover and d) elevation of spotted nutcracker caching and harvesting sites. Boxes indicate the 25% and 75% quartiles, black line indicates the median. Whiskers extend to the highest value that is within 1.5 × the interquartile range. Grey background color in d) indicates elevational range (1850–2150 m a.s.l.) of Swiss stone pines in the study area

The space used by individual nutcrackers at harvesting sites was significantly smaller than at caching sites (Fig. 2b; Estimate: − 13.4, SE 3.43, t = − 3.8, *p* = 0.003). While harvesting areas were on average 11 ha in size, caching areas were 25 ha in size. The straight-line distance between harvesting and caching sites ranged from 2.1 to 8.5 km with a mean of 5.2 km between sites. Importantly, harvesting sites were shared among most individuals, which may have been an artifact of selecting a forest with high nutcracker activity as capture location; however, caching sites were spatially dispersed and not shared (Fig. 1).

There was a significant difference in pine forest cover overlap between harvesting and caching sites (Fig. 2c, Estimate: 68.9, SE 8.3, t = 8.3, *p* < 0.0001). While harvesting sites overlapped on average 80% with pine forest (i.e., with forest that contained a minimum of 10% Swiss stone pine tree cover), there was only 11% overlap with pine forest at caching sites. Harvesting sites were located at higher elevations than caching sites (Fig. 2d, Estimate: 244.0, SE 45, t = 5.4, *p* < 0.0002). While harvesting sites were located at on average 2047 m a.s.l., caching sites were located at 1803 m a.s.l. and thus, mostly outside the known range of pine occurrence in the study area (i.e., between 1850 and 2150 m a.s.l.).

## Discussion

Here, we provide novel insights into the seed dispersal potential of a scatter hoarding bird using spatially and temporally detailed GPS movement data. Our results show routine individual daily movements between a single harvesting and a single caching site, shared harvesting sites but separate caching sites among individuals, and long-distance seed dispersal events. Expected harvesting and caching sites were clearly distinguishable by nutcracker visit duration, area, pine forest cover, and elevation. Interestingly, our results suggest that spotted nutcrackers cache seeds mostly at low elevations, outside the known range of occurrence of Swiss stone pine in the study area. This suggests that nutcrackers disperse seeds predominantly to sites with a low probability of pine regeneration. However, caching areas were much larger than foraging areas, which could increase the chances of future recruitment even at marginal densities.

Nutcrackers routinely dispersed seeds over very long distances (up to 8.5 km straight line path in this study). These directional dispersal movements were made on a remarkably regular basis, which is why they may not meet the classical definition of long-distance seed dispersal (i.e., mostly referring to rare and extreme events (see e.g., [13, 32]). Compared to previous studies, the seed dispersal distances observed in this study were beyond what has usually been recorded for many plant species (e.g., [41]). Even scatter-hoarding birds, such as jays or scrub-jays, which are usually very effective seed dispersers, mostly disperse seeds over much smaller distances (reviewed in [37]). Only previous studies on Clark’s nutcrackers (*Nucifraga columbiana*) in North America, have shown exceptional seed dispersal flights up to 30 km [25, 26, 37].

In our study, sites chosen for seed caching were located at low elevations outside the elevational range of the pine. In another scatter-hoarding system, European jays transport seeds from oak to pine stands which is considered beneficial for the viability of oak populations and the diversity of overall forest structure [17]. We find a similar pattern, dispersal between areas dominated by different tree species,however, seed dispersal to spruce forests is likely not very beneficial for the pine, as pine is outcompeted by spruce at its lower elevational range edge [28, 42]. Previous work has further shown that the conditions for pine recruitment are very poor at the lower elevational range of the species, mainly due to high rates of seed predation [28, 33]. For seeds that are dispersed to sites at elevations lower than the lower elevational range edge, it seems that recruitment success is even more limited, which is why we assume that long-term establishment and growth hardly occurs in spruce-dominated habitat. However, we emphasize that even rare success in recruitment would be sufficient to maintain population viability [10], in particular given the long life-span of Swiss Stone pine [34]. The fact that caching areas were much larger than foraging areas further increases the spatial dispersion of the species, even though recruitment success likely happens rarely at caching sites. Clark’s nutcrackers were also found to cache seeds at low elevations, probably to avoid a thick snow layer in winter [26]. We expect a similar motivation for spotted nutcrackers as lower elevation sites with benign abiotic conditions are preferable breeding territories. Additionally, spotted nutcrackers are known to select microsites which promote long term storage of seeds rather than sites that are optimal for pine seed germination [34]. Taken together, this suggests that spotted nutcrackers may not offer effective long-distance seed dispersal for Swiss stone pines.

Although our results suggest that effective long-distance seed dispersal in the spotted nutcracker and Swiss stone pine system does not occur en masse in a typical year, masting years and rare seed dispersal events may result in sufficiently effective seed dispersal for maintaining population viability. Masting years, cyclical and spatially synchronized bumper crops [24], may cause consumer populations to be overwhelmed resulting in a larger number of unrecovered seeds that survive until reproduction [18, 22]. However, the specific effects of masting on scatter-hoarding behavior of corvids are not fully understood and require further study [37]. Rare seed dispersal events may also make up for the unfavorable caching sites observed in this study. Rare events, occurring in less than 1% of seed dispersal events, may be sufficient to maintain genetic links between subpopulations and to allow for regeneration of pine populations. It has been shown previously that rare long-distance dispersal events may be disproportionately important for plant fitness and migration rates [9, 31]. For instance, movements of juvenile nutcrackers during dispersal to distant breeding sites could result in rare long-distance seed dispersal events, as observed in the Sardinian warbler (*Sylvia melanocephala,*[16]. Little is known about whether the timing of juvenile dispersal occurs during the cone ripening and harvesting season, or if such movements could result in seed dispersal to sites beneficial for pine germination and recruitment. The potential role of rare long-distance dispersal events not observed in this study, including the possibility of those carried out by juveniles moving to future breeding territories, are important areas of future research.

We found that the time spent at expected harvesting sites was greater than the time spent at expected caching sites, likely due to the greater time required to harvest vs. cache seeds [25]. For instance, Mattes [27] showed that a nutcracker needs about 33 s to establish one seed cache, whereas, removing seeds one at a time from the hard pine cone is more time consuming. These results suggest that movement data alone may be used to distinguish between spotted nutcracker harvesting and caching sites. It is important to note that previous studies have found that caching behaviour also occurs at our main harvesting site (Flüela valley; [28, 33], which may contribute to the greater time spent there. Such caching behaviour could be long term, or simply a temporary storage option [27] before most seeds are transported to distant lower elevation caching sites. Indeed, local seed dispersal at the harvesting site is important for Swiss stone pine recruitment across, and, in particular, at the upper elevational range limit of the pine [33]. Future movement studies of scatter-hoarding species may wish to make use of similar characteristics of movement data to distinguish between harvesting and caching sites. Possibilities for using the time spent in distinct locations as indicators of the activities carried out is more challenging for non-scatter hoarders but could include investigations into roosting and resting times [23], or certain habitat characteristics in combination with the duration of movement behaviours.

## Conclusions

In conclusion, spotted nutcrackers may provide poor long-distance seed dispersal service for Swiss stone pines as seeds are mainly dispersed beyond the lower elevational edge of the pine distribution. This dispersal pattern will be particularly challenging for the pine in the course of ongoing climate change as plants are likely to shift their occurrence to higher elevations [44]. However, future studies will be required to improve our understanding of the nutcracker’s contribution to pine forest rejuvenation, including an extensive tracking program during a Swiss stone pine masting year.

## Supplementary Information

**Additional file 1**. Containing figures S1 and S2, showing revisitation densities of all tagged birds and movements of one juvenile bird, respectively.

## Data Availability

The GPS data supporting the conclusions of this research will be made available on movebank.org.
