# Supplementary material for: Scatter-hoarding birds disperse seeds to sites unfavorable for plant regeneration
Source: Mov Ecol. 2022 Sep 17;10:38. doi: 10.1186/s40462-022-00338-1 (PMC9482738; doi:10.1186/s40462-022-00338-1)
Supplement: Supplementary file 1 — Additional file 1. Containing figures S1 and S2, showing revisitation densities of all tagged birds and movements of one juvenile bird, respectively. [file 40462_2022_338_MOESM1_ESM.docx]

**Supplementary material for:** *Scatter-hoarding birds disperse seeds to habitats unsuitable for plant regeneration*. Marjorie C. Sorensen, Thomas Müller, Isabel Donoso, Valentin Graf, Dominik Merges, Marco Vanoni, Wolfgang Fiedler, Eike Lena Neuschulz.

Fig. S2. Map of the Flüela valley (4648’0.25”N, 954’15.38”E) near Davos, Switzerland, showing movements of one juvenile spotted nutcracker over 20 days during the 2017 harvesting season. Blue points show each GPS location. Green areas show forest with more than 10% Swiss stone pine cover.

Fig. S1. Revisitations of spotted nutcrackers to a 500 m radius around each GPS point location, depicted as the revisitation density in relation to the percentage of maximum revisits. The red line indicates the threshold between rarely and frequently revisited sites with T indicating the exact threshold value. The individual tag ID is given on top of each panel. Note that the radius for revisitation was 300 m for tag # 20.
